# Dynamic Identification Method for Highway Subgrade Soil Compaction Based on Embedded Attitude Sensors

**DOI:** 10.3390/ma18204801

**Published:** 2025-10-21

**Authors:** Zhizhou Su, Hao Li, Jiaye Hu, Bin Wu, Fengteng Liu, Peixin Tian, Xukai Ding

**Affiliations:** 1 School of Instrument Science and Engineering, Southeast University, 2 Sipailou, Nanjing 210096, China; suzz_mail@seu.edu.cn (Z.S.); hujiaye5@163.com (J.H.); 2 JSTI Group Co., Ltd., Nanjing 210019, China; lihao@jsti.com; 3 Nanjing Digintec Development Co., Ltd., Nanjing 211112, China; wb192@jsti.com; 4 School of Transportation, Southeast University, 2 Sipailou, Nanjing 210096, China; 220233397@seu.edu.cn (F.L.); 220233446@seu.edu.cn (P.T.)

**Keywords:** subgrade compaction, embedded attitude sensor, inertial measurement unit (IMU), signal processing, XGBoost

## Abstract

Compaction quality is a critical factor in ensuring the long-term performance of subgrade structures; however, traditional testing methods are limited by their destructive nature and delayed feedback. To address these shortcomings, this study proposes a dynamic identification method for subgrade compaction based on embedded attitude sensors. A customized sensor unit integrated with an inertial measurement module was embedded in soil samples to record triaxial acceleration and attitude angles during the compaction process. Signal processing techniques, including an improved wavelet-based denoising strategy, were employed to separate long-term compaction trends from transient impact disturbances. Attitude features such as cumulative angular change, angular velocity, root mean square values, and a comprehensive inclination index were extracted as predictive variables. Ridge regression, random forest, and XGBoost models were constructed to establish the mapping relationship between attitude features and compaction degree. Experimental results on clay, loam, and sand samples indicate that the yaw angle is most sensitive to vertical settlement, while pitch and roll angles provide complementary information on lateral and rotational behaviors. Comparative analysis of filtering methods shows that the transient masking interpolation (TMI) approach outperforms the traditional asymmetric wavelet thresholding (AWT) method in effectively preserving baseline trends. Among the regression models, XGBoost demonstrated the best predictive performance, achieving an R^2^ exceeding 0.995 at high compaction levels. The proposed method has been experimentally demonstrated as a laboratory-scale proof of concept, showing strong potential for future real-time field application, offering a novel technological pathway for intelligent quality control in road construction.

## 1. Introduction

Compaction is a crucial process in road construction to ensure the quality of subgrade and pavement structures. Proper compaction can significantly improve soil stiffness, strength, and overall stability, thereby enhancing the service performance and durability of pavement structures [1,2,3]. However, achieving the efficient, accurate, and comprehensive monitoring of compaction quality during construction has long been a key challenge in highway construction and maintenance. Traditional quality inspection methods mainly rely on post-construction on-site sampling, which represents “point control” and “after-the-fact control.” These methods are random and lagging, often missing localized areas of insufficient compaction, and once problems are identified, the optimal time for corrective rolling is usually lost [4]. In addition, common methods such as the cutting ring and sand cone tests are destructive, causing damage to the completed subgrade and compromising overall construction quality [5,6]. These drawbacks indicate that traditional methods can no longer meet the modern demands of highway engineering for high efficiency and high reliability.

To overcome the limitations of traditional methods, various technical explorations have been carried out in recent years. Choi et al. developed a novel nondestructive time-domain reflectometry (TDR) system integrated with piezoelectric stacks [7]. The system adopts a flat-plate probe design, enabling rapid measurement of dry density, water content, and elastic modulus on compacted soil surfaces without disturbing the soil. Validation tests on four types of sandy soils demonstrated that the measured dry density and water content showed good agreement with the results of standard compaction tests, thereby providing a new approach for rapid nondestructive evaluation of compaction quality. For the assessment of surface compaction in difficult-to-compact sandy subgrades, Tymosiak et al. conducted calibration chamber experiments using a lightweight dynamic cone penetrometer (DCP). By obtaining the penetration index (PI) and the number of blows required for 10 cm penetration (N_10_(DCP)), they established a prediction model of compaction index (IS) as a function of test depth z and N_10_(DCP) [8]. The results confirmed the feasibility of the DCP probe for indirect evaluation of surface compaction quality in non-cohesive subgrades; however, the method requires separate calibration for different gradations of non-cohesive soils, limiting its general applicability. Building upon these efforts, continuous compaction control (CCC) technology has emerged to further enable real-time monitoring of the compaction process [9,10]. This approach installs sensors on compaction equipment to monitor the roller’s dynamic response in real time and evaluates subgrade compaction levels based on the correlation between the response signals and compaction quality [11,12,13]. As early as the 1970s, Sweden introduced the concept of a compaction meter, developing devices that could be mounted on rollers and display compaction quality via a dial indicator, leading to the most classic compaction index—the Compaction Meter Value (CMV), defined as the ratio of the second harmonic amplitude to the fundamental amplitude in the acceleration signal of the vibrating drum. Studies have shown that CMV has a certain correlation with conventional compaction quality indicators such as modulus and degree of compaction [14,15,16]. Due to its clarity and ease of implementation, CMV is still widely applied in both research and practice of CCC [17,18]. However, CMV has notable limitations: first, when soil stiffness is high, resonance and drum bouncing can occur, reducing CMV and weakening its correlation with actual compaction quality [19,20]; second, CMV considers only the second harmonic component in the acceleration spectrum, whereas the real spectrum contains many harmonics, making it difficult to ensure accuracy with a single component. To address this, researchers have proposed improved indices such as CCV and THD by incorporating more spectral components [21]. For example, Sakai in Japan proposed CCV, which takes into account multiple harmonics and subharmonic components, while Mooney et al. [22] introduced THD, defined as the ratio of the root mean square of higher-order harmonics to the fundamental amplitude. Nonetheless, the relationship between these harmonic ratio indices and compaction quality largely relies on empirical observations and lacks systematic theoretical support, which to some extent limits their further development and application [23].

To reveal the physical mechanisms of harmonic and subharmonic components in acceleration signals, previous studies have employed finite element methods to numerically simulate the vibratory compaction process, investigating the effects of geometric nonlinearity, contact nonlinearity, and material nonlinearity on signal distortion [24]. The results show that material nonlinearity and contact nonlinearity are major sources of harmonic components, while contact nonlinearity induced by drum bouncing is the fundamental cause of subharmonic components [9]. These studies theoretically explain the origins of spectral components and provide support for the scientific development of harmonic ratio-based compaction indices. However, from a practical perspective, continuous compaction control primarily monitors the roller’s dynamic response, which essentially reflects “machine response” rather than the true deformation and energy dissipation of the soil. This indirectness makes the results highly susceptible to the combined influence of construction equipment, process parameters, and soil conditions, thus limiting the accuracy and applicability of monitoring.

It is noteworthy that in the field of asphalt mixtures, which are closely related to subgrade performance, researchers have increasingly recognized the decisive role of the material microstructure in governing its macroscopic properties. Through multiscale analysis and precise monitoring techniques, valuable insights have been provided to overcome the limitations of “indirect monitoring.” Peng et al. [24] systematically investigated the influence of aggregate morphology and spatial distribution on the mechanical response and pavement performance of asphalt mixtures through macro- and meso-scale multiscale analyses. The results showed that the microscopic characteristics of aggregates significantly alter the internal stress–strain distribution of the mixture. Neglecting aggregate effects leads to underestimation of the actual strain level, while a uniform spatial distribution of aggregates can form a stable load-bearing skeleton, reducing stress transfer to the mortar phase. This theoretically confirmed that only by capturing the internal microstructural state of materials can their macroscopic performance be accurately evaluated. Building on this, Ding et al. [25] further focused on the microscale monitoring of aggregate skeletons in asphalt mixtures. Based on sphere-packing theory, they proposed a weighted average particle size criterion for gradation design and innovatively embedded Smart Rock (SR) sensors into aggregate skeletons. By monitoring motion indicators such as triaxial acceleration and angular acceleration of the sensors, they directly captured aggregate sliding and rearrangement processes, verifying the feasibility of embedded sensors in monitoring skeleton stability. Their study also revealed that, compared with conventional stress–strain indices, micro-motion data such as acceleration and angular acceleration more accurately reflect the damage evolution of aggregate skeletons. This provides a novel technical pathway for direct monitoring of the internal state of materials. However, the above experiments were only conducted on asphalt mixture materials, and lack validation in the context of subgrade soils.

In this context, it is urgent to develop a novel approach that can directly reflect the internal compaction state of subgrade soils. This paper proposes a dynamic identification method for highway subgrade soil compaction based on embedded inertial sensors. Small attitude sensors are buried within the soil to record the triaxial acceleration and attitude changes in the soil during construction in real time. Through signal processing and feature extraction, a mapping relationship is established between the sensor response and compaction quality. Compared with traditional methods, this approach avoids the lag of “post-construction testing” and the indirectness of “machine-mounted monitoring,” enabling real-time, continuous, and nondestructive evaluation of the compaction process. Its key advantage lies in capturing the full process of internal soil dynamic response and structural evolution, thus providing new technical support for dynamic compaction identification and intelligent construction control, as well as offering new possibilities for improving the scientific rigor and precision of highway subgrade compaction quality monitoring.

In this study, an embedded attitude sensor with a glass fiber–reinforced nylon package was selected as the core sensing unit. By combining theoretical analysis with experimental investigation, the evolution characteristics of soil attitude inside the subgrade under varying compaction levels were examined. The novelty of this study lies not only in the application of IMUs but also in the development of a survivability-verified embedded sensor that operates reliably under high-pressure compaction environments. Furthermore, the study establishes an integrated framework coupling wavelet-based signal denoising, physics-aware feature engineering, and AI-based modeling to dynamically identify soil compaction behavior. This approach bridges the gap between traditional geotechnical monitoring and intelligent data-driven analysis, enabling new possibilities for real-time, in situ pavement quality control. The research aims to explore the influence mechanism of compaction on pose parameters based on Euler angles under subgrade soil conditions, and to analyze the correlations between fundamental pose quantities, their derived parameters, and pavement compaction degree. This work provides technical support for applying embedded attitude sensors to monitor the internal state of subgrade soils.

## 2. Materials and Methods

### 2.1. Design and Principle of the Embedded Attitude Sensor

Traditional compaction monitoring methods largely rely on measurements at the surface of compaction machinery or on discrete soil sampling, lacking sensors capable of being embedded within the soil and stably operating under high-frequency, high-pressure loading conditions. Conventional inertial or pressure sensors often fail to survive in such harsh environments, particularly due to their inability to continuously output valid data from within the material. Therefore, building upon existing inertial measurement and attitude estimation techniques, this study developed an embedded attitude sensor specifically designed for soil compaction processes, and verified its stability and feasibility under severe compaction conditions.

**Figure 1:**
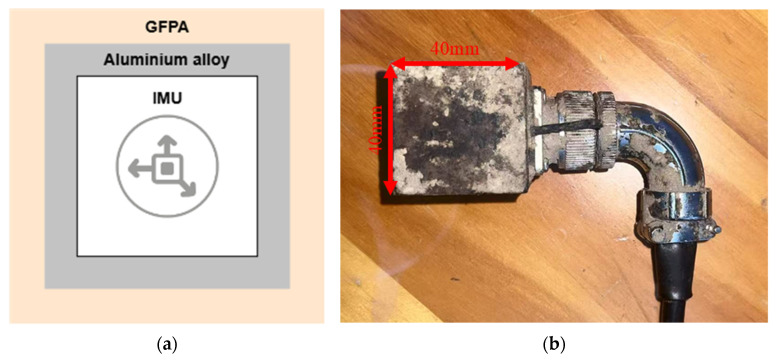
(**a**) Structure of the embedded attitude sensor; (**b**) photograph of the embedded attitude sensor.

The device is centered on an inertial measurement unit (IMU) and its control circuitry (Figure 1a), capable of continuously acquiring triaxial acceleration and magnetic field data at a sampling frequency of 100 Hz. Through attitude estimation algorithms, it provides real-time outputs of pitch, roll, and yaw angles, enabling continuous dynamic identification of soil compaction from within the soil mass.

To ensure functional integrity under high-frequency impacts and high-pressure loads, the sensor adopts a dual-layer cubic packaging structure: an internal aluminum alloy framework that provides sufficient stiffness and resistance to deformation, and an external glass-fiber reinforced nylon housing that transmits external loads while serving as a buffer layer to mitigate impact stresses on the core components. The overall packaging takes the form of a cube with 4 cm side length, which ensures stable embedding within soil samples while balancing protection and operability, as shown in Figure 1b. The signal output is achieved via a 7 mm-diameter cable using an RS-485 bus transmission protocol, enhancing anti-interference capability and improving data transmission stability. Data are finally collected and stored in a host computer for unified processing.

The data transmitted from the embedded attitude sensor consist of the measured acceleration and magnetic field directions rather than direct pose information. Therefore, an attitude calculation algorithm must be applied to the acceleration and magnetic data to obtain the actual attitude parameters, namely pitch, roll, and yaw. First, the triaxial components of acceleration and magnetic field are read and normalized:

$$a^{b}=\left[\begin{array}{c} a_{x}\\ a_{y}\\ a_{z} \end{array}\right],m^{b}=\left[\begin{array}{c} m_{x}\\ m_{y}\\ m_{z} \end{array}\right]$$

$${\hat{a}}^{b}=\frac{a^{b}}{\left\|a^{b}\right\|},{\hat{m}}^{b}=\frac{m^{b}}{\left\|m^{b}\right\|}$$

The roll angle (ϕ) and pitch angle (θ) are determined based on the gravity vector. After compensating for the effects of pitch and roll, the yaw angle (ψ) is subsequently calculated using the geomagnetic vector.

$$\phi =arctan\left(a_{y},a_{z}\right)$$

$$\theta =arctan\left(-a_{x},\sqrt{a_{y}^{2}+a_{z}^{2}}\right)$$

$$\begin{array}{l} m_{x}^{c}=m_{x}cos\theta +m_{z}sin\theta \\ m_{y}^{c}=m_{x}sin\phi sin\theta +m_{y}cos\phi -m_{z}sin\phi cos\theta \end{array}$$

$$\psi =arctan\left(-m_{y}^{c},m_{x}^{c}\right)+\delta_{D}$$

where $\delta_{D}$ denotes the magnetic declination.

### 2.2. Subgrade Soil Preparation and Standard Compaction Degree Calculation

**Figure 2:**
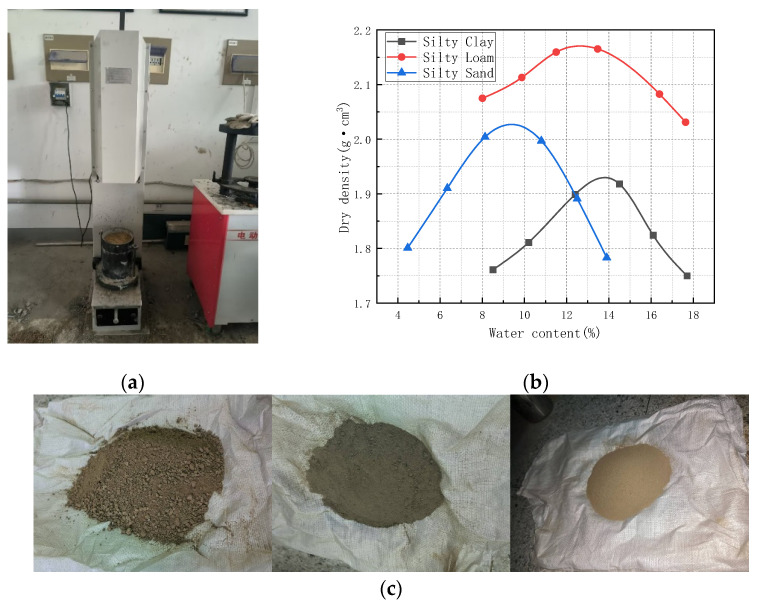
Preparation of soil samples for the compaction test. (**a**) Field compaction test; (**b**) relationship curve between dry density and water content; (**c**) photographs of the three soil types, from left to right: clay, loam, and sand.

According to the Test Methods of Soils for Highway Engineering (JTG 3430-2020) [26], three types of subgrade soils from cohesive to frictional were prepared: silty clay, silty loam, and silty sand. Physical properties of these types of soil are shown in Table 1. For each soil type, six specimens with different water contents were prepared by increasing the moisture content in increments of 2%. The specimens were compacted in a cylindrical mold with an inner diameter of 15.2 cm and a height of 17 cm using the heavy compaction method II with a three-layer procedure. The test results are shown in Figure 2. For silty clay, the maximum dry density ($\rho_{dmax}$) was 1.93 g/cm^3^, and the optimum water content ($w_{0}$) was 13.7%. For silty loam, the maximum dry density ($\rho_{dmax}$) was 2.17 g/cm^3^, and the optimum water content ($w_{0}$) was 12.6%. For silty sand, the maximum dry density ($\rho_{dmax}$) was 2.03 g/cm^3^, and the optimum water content ($w_{0}$) was 9.4%.

**Figure 3:**
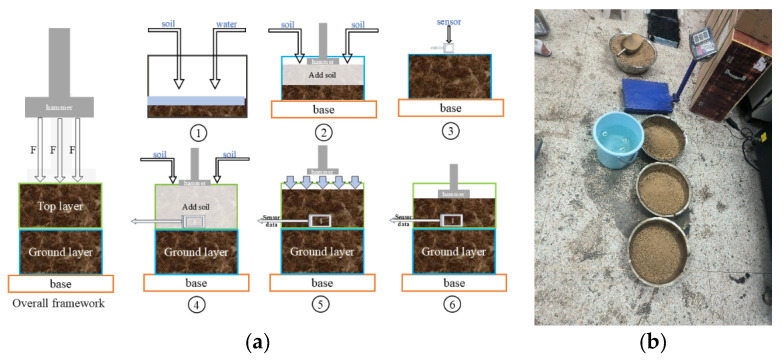
(**a**) Flowchart of the experimental design for sensor monitoring (cross-sectional view). ① Soil pretreatment and water mixing; ② padding soil into the large Marshall mold with slight pre-compaction; ③ placement of the sensor; ④ filling the remaining soil and pre-compaction; ⑤ compaction until no further settlement of the soil layer occurs; ⑥ data recording and storage. (**b**) Experiment site.

Compaction tests were carried out using large Marshall specimens. First, the prepared soil samples were thoroughly mixed with water and placed into the large Marshall mold up to half of its height. The sensor was then embedded at the mid-layer of the soil, with the cable led out through a customized perforated upper mold to ensure that signal transmission was not affected by the compaction process. After filling the remaining soil and performing pre-compaction, the specimen was compacted manually until no further settlement of the soil layer occurred. The entire experimental procedure is illustrated in Figure 3a, comprising six steps: soil mixing with water, pre-compaction, sensor embedding, upper soil filling, compaction, and data recording. The actual experimental setup is shown in Figure 3b, which provides a clear visualization of the specimen formation and the full data acquisition process. For each soil type, three independent compaction experiments were conducted under identical boundary conditions. Each test yielded 4000–10,000 synchronized readings of attitude, and compaction degree during compaction is recorded through real-time video, ensuring adequate data volume and statistical repeatability for reliable model development.

For the calculation of soil compaction degree ($c_{soil}$) in the experiment, the following formula is used:

$$c_{soil}=\frac{\rho }{\rho_{s}}$$

where $\rho =\frac{m_{soil}}{V_{m}}$ represents the real-time density, $\rho_{s}$ is the standard density of the soil type, $m_{soil}$ is the total mass of soil in the mold, and $V_{m}$ is the real-time volume of the specimen.

Based on this formula, the real-time compaction degree of the Marshall specimen can be calculated from the instantaneous settlement at the top surface, which can then be correlated with the internal attitude data simultaneously collected by the embedded attitude sensor, thereby establishing the relationship between the two.

This section introduces the setup of the experimental environment and the preparation of experimental materials. On this basis, the embedded attitude sensor was employed to continuously acquire real-time internal attitude changes in the soil samples within the Marshall mold, while the real-time compaction degree was recorded in parallel. These synchronized datasets provide the foundation for subsequent feature analysis and the establishment of coupled predictive models.

Based on the above steps, the embedded attitude sensor enables real-time acquisition of internal attitude changes within the soil specimen in the large Marshall mold, while simultaneously recording real-time compaction degree. This provides a fundamental dataset for subsequent feature analysis and the development of coupled models.

### 2.3. Attitude Slow-Varying Signal Extraction Method Based on an Improved Wavelet Denoising Strategy

Euler angles (pitch, roll, and yaw) were adopted due to their clear physical interpretation in engineering contexts. Although Euler angles can be sensitive to noise, this limitation was effectively mitigated through multi-level wavelet-based denoising, which preserved physical features while suppressing high-frequency noise components. Prior to model training, attitude signals were preprocessed using an 8-level discrete wavelet transform (sym4 basis) for denoising. Subsequently, multiple dynamic and statistical features—including angular velocity, RMS, variance, and composite attitude magnitude—were extracted to form the machine learning inputs. During the subgrade soil compaction process, the Euler angle signals acquired by the embedded attitude sensor typically consist of three distinct components: (i) a slow-varying trend induced by the progressive densification of the soil, which accurately characterizes the actual compaction process, (ii) pulse-like disturbances arising from the instantaneous impact of the rammer, superimposed with environmental noise, and (iii) zero-mean environment noises. Therefore, it is necessary to preprocess the signals to separate the effective trends from transient disturbances and to extract low-frequency variations that can reflect changes in soil compaction.

Let the pose variation signal caused by compaction be denoted as $\Theta \left(t\right)$, which can be decomposed into three parts:

$$\Theta \left(t\right)=\Theta_{s}\left(t\right)+\Theta_{tr}\left(t\right)+\epsilon (t)$$

Here, $\Theta_{s}\left(t\right)$ denotes the slow-varying attitude curve formed by the cumulative effect of progressive compaction, which is the primary focus of this study; $\Theta_{tr}(t)$ represents the transient disturbances induced by rammer impacts, characterized by a non-zero mean; and ϵ(t) corresponds to environmental noise with an approximately zero mean. The objective of the data preprocessing procedure is to effectively suppress $\Theta_{tr}(t)$ and ϵ(t) while preserving $\Theta_{s}(t)$.

Transient disturbance detection was first carried out using the Stationary Wavelet Transform (SWT) for multi-scale decomposition. Considering that the primary energy of the rammer impact is concentrated within the frequency range of 2–30 Hz, the corresponding sets of scale coefficients were selected for energy envelope calculation.

$$E\left(t\right)=\sqrt{\sum_{j\in J}{\left(d_{j}^{+}(t)\right)}^{2}}+\delta$$

Here, $d_{j}^{+}\left(t\right)$ denotes the positive half-wave coefficients at scale j, and δ is a small bias term introduced to suppress minor jitter. Subsequently, the Median Absolute Deviation (MAD) method was employed for threshold estimation, and morphological closing operations were applied to smooth the energy envelope, thereby identifying the transient disturbance interval Ω.

For filtering disturbance signals within the identified transient intervals, two strategies can be adopted. The first is Asymmetric Wavelet Thresholding (AWT), a local impact suppression algorithm based on asymmetric attenuation of wavelet-domain coefficients. This method modifies the coefficients at different decomposition levels and applies one-sided suppression specifically to those within the vibratory compaction frequency range.

$${\tilde{d}}_{j}\left(t\right)=\left\{\begin{array}{c} sgn\left(d_{j}(t)\right)\cdot \max\left(\left|d_{j}\left(t\right)\right|-\lambda_{j}^{+},0\right),\;\;\;\;\;d_{j}\left(t\right)>0\\ sgn\left(d_{j}(t)\right)\cdot \max\left(\left|d_{j}\left(t\right)\right|-\lambda_{j}^{-},0\right),\;\;\;\;\;d_{j}\left(t\right)<0 \end{array}\right.$$

Here, $\lambda_{j}^{+}$ and $\lambda_{j}^{-}$ denote the thresholds in the positive and negative directions, respectively. When the impact is primarily manifested as a positive pulse, the condition $\lambda_{j}^{+}>\lambda_{j}^{-}$ can be applied to achieve stronger suppression of the transient components on the positive half-axis, and vice versa. The reconstructed signal can then be expressed as:

$$\hat{\Theta }\left(t\right)=a_{J}\left(t\right)+\sum_{j=1}^{J}{\tilde{d}}_{j}(t)$$

This method is based on the concept of one-sided wavelet coefficient correction, which preserves the overall trend while simultaneously suppressing local impacts.

The Asymmetric Wavelet Thresholding (AWT) method is an algorithm that directly masks the transient intervals and reconstructs the signal through weighted smoothing spline interpolation. Its computational formulation is given as:

$$\hat{\Theta }\left(t\right)=\arg\underset{\Theta }{\min}\sum_{t\notin \Omega }{\left(\Theta \left(t\right)-\Theta_{s}\left(t\right)\right)}^{2}+\lambda {\left|\left|\Delta^{2}\Theta (t)\right|\right|}^{2}\;$$

Here, Ω denotes the transient interval, $\Delta^{2}$ represents the second-order difference operator, and λ is the smoothing parameter controlling the degree of smoothness.

To evaluate the effectiveness of the filtering process, both the Root Mean Square Error (RMSE) and smoothness were employed for comprehensive analysis. RMSE measures the proximity of the filtered signal to the original signal, while smoothness reflects the extent to which high-frequency components have been effectively removed. Since the primary objective of filtering is to restore the overall slow-varying trend of the subgrade soil during compaction, the ideal filtering outcome should achieve the smallest possible smoothness while maintaining an RMSE value that does not deviate excessively. The calculation methods for these two indices are given as follows:

$$RMSE=\sqrt{\frac{1}{N}\sum_{i=1}^{N}{\left({\hat{x}}_{i}-x_{i}\right)}^{2}}$$

$$Smoothness=Var({\left\{{\hat{x}}_{i+1}-{\hat{x}}_{i}\right\}}_{i=1}^{N-1})$$

Meanwhile, the difference in smoothness between the filtered signal and the original signal was computed to obtain ΔSmoothness. A larger absolute value of this metric indicates a better filtering performance. The root mean square error (RMSE) should be evaluated in conjunction with the baseline trend of the waveform before and after filtering. If the baseline trend is preserved and the RMSE does not exhibit a significant increase, it can be considered that the baseline has not been substantially shifted after filtering, thereby indicating that the filtering method is acceptable.

### 2.4. Design of Derived Features Based on Fundamental Attitude Quantities

After the filtering process, the attitude angle signals can more stably reflect the overall trend of the compaction process. However, to establish a relationship between these raw angular data and the degree of compaction, it is necessary to extract a set of indices that characterize the dynamic features of compaction. Based on the three-dimensional attitude angles—Pitch(θ), Roll(ϕ), and Yaw(ψ)—several feature quantities were designed and calculated. These features not only capture the temporal variation patterns of the attitude signals but also possess clear engineering and physical significance.

The following feature quantities were selected for subsequent feature analysis and model construction:

Primary features:

θ(t),ϕ(t),ψ(t)

Relative to the raw angular values, this feature reflects the overall displacement of the sensor attitude within a compaction cycle, thereby indicating whether the soil undergoes sustained settlement or tilting under the compaction process.

$$\Delta \theta =\theta \left(t\right)-\theta \left(t_{0}\right),\;\Delta \phi \left(t\right)=\phi \left(t\right)-\phi \left(t_{0}\right),\;\Delta \psi =\psi \left(t\right)-\psi (t_{0})$$

By applying a first-order difference to the attitude angles, the angular velocity features can be obtained (similarly for ϕ and ψ). Angular velocity characterizes the instantaneous rate and intensity of response during the compaction process, where abrupt changes are often associated with hammer impacts or rapid adjustments in the soil structure.

$$\dot{\theta }\left(t\right)\approx \frac{\theta \left(t\right)-\theta \left(t-\Delta t\right)}{\Delta t}$$

A composite angular velocity magnitude can be further constructed to provide a quantitative measure of the overall rate of attitude variation.

$$\Omega \left(t\right)=\sqrt{\theta {\left(t\right)}^{2}+\phi {\left(t\right)}^{2}+\psi {\left(t\right)}^{2}}$$

To characterize short-term variations in attitude intensity, a windowed Root Mean Square (RMS) feature was introduced to represent the activity level of attitude changes within a short time window N (similarly for ϕ,ψ). This feature can be interpreted as a local energy indicator, providing a measure of the compaction intensity of the soil over a specific time interval.

$$RMS_{\theta }\left(t\right)=\sqrt{\frac{1}{N}\sum_{k=t-N/2}^{t+N/2}\dot{\theta }{\left(k\right)}^{2}}$$

In addition to dynamic variations, the combined relationship of attitude angles can also reflect the uniformity of compaction. Therefore, a biaxial local inclination index was defined. Taking the combination of pitch and yaw as an example, this index characterizes the magnitude of sensor displacement in the Pitch–Yaw plane, where larger values indicate stronger lateral displacement of the soil in that region.

$$T\left(t\right)=\sqrt{\theta {\left(t\right)}^{2}+\psi {\left(t\right)}^{2}}$$

In addition, combinations of roll–pitch and roll–yaw angles were also incorporated to ensure comprehensiveness in the analysis. Furthermore, a sliding-window-based local angular variance was introduced to quantify the short-term fluctuations of attitude angles within the window (similarly for ϕ,ψ). A larger variance indicates significant oscillations in the attitude curves, which may be associated with local inhomogeneities in the soil structure.

$$Var_{\theta }\left(t\right)=\frac{1}{N}\sum_{k=t-N/2}^{t+N/2}{\left(\theta \left(k\right)-\bar{\theta }\right)}^{2}$$

In summary, the aforementioned features characterize the dynamic response of sensor attitude during the compaction process from multiple perspectives, including cumulative variation, instantaneous rate, overall intensity, and local uniformity. From an engineering standpoint, cumulative variation reflects the overall compaction trend, angular velocity and RMS indices capture the intensity and energy level of the compaction process, and inclination and local variance reveal the stability and uniformity of the soil structure. The comprehensive extraction of these features provides a more discriminative set of input variables for subsequent regression modeling.

### 2.5. Data Regression Algorithms Based on Ridge Regression, XGBoost, and Random Forest

After feature extraction, it is necessary to establish a mapping relationship between the attitude features and the compaction degree in order to achieve a quantitative assessment of the construction state. In this study, three representative regression methods were selected: Ridge Regression (RR), XGBoost regression, and Random Forest (RF). These methods, respectively, correspond to linear modeling, nonlinear boosted trees, and ensemble learning, thereby capturing the influence of attitude features on compaction degree from different perspectives. To ensure model reliability and reproducibility, a rigorous validation strategy was implemented. The complete dataset was randomly divided into 70% for training and 30% for testing, and this partitioning was repeated across five random seeds to minimize selection bias. Within the training subset, a 5-fold cross-validation procedure was conducted to evaluate model generalization and stability. The performance of each model was quantified using both test-set and cross-validation metrics, including RMSE and R^2^, and the mean ± standard deviation across folds (CV_RMSE, CV_R^2^) are reported. This dual-level validation design provides a statistically consistent evaluation of the predictive framework.

To unify data input, let the sampling interval be denoted as Δt. At a given time point $t_{i}=i\Delta t$, the corresponding attitude angles and compaction degree are expressed as follows:

$$\Theta_{i}=\left(\theta_{i},\phi_{i},\psi_{i}\right),p_{i}=p(t_{i})$$

The compaction degree time series was aligned to the attitude time axis via spline interpolation, resulting in a unified index set $\{p_{i}{\}}_{i=1}^{N}$. Subsequently, the features defined in the previous section were computed to obtain the feature vector $x_{i}$, with $y_{i}=p_{i}$ serving as the supervisory signal, thereby forming the sample set ${\left\{\left(x_{i},y_{i}\right)\right\}}_{i=1}^{N}$. To ensure the validity of all formulations, nearest-neighbor extension was applied at the window boundaries.

#### 2.5.1. Ridge Regression

Ridge Regression is a linear regression approach with $l_{2}$-regularization, designed to mitigate the adverse effects of multicollinearity among features on model stability. In this study, the ridge regression model was employed to directly estimate the linear response of compaction degree to attitude angles and their short-term statistical features. The model can be expressed as

$$y^{i}=\beta_{0}+\beta^{⊤}x_{i},\underset{\beta 0,\beta }{\min}\sum_{i=1}^{N}{\left(y_{i}-\beta_{0}-\beta^{⊤}x_{i}\right)}^{2}+\lambda {\left|\left|\beta \right|\right|}_{2}^{2}\;$$

Let $X=\left[x_{1}^{⊤};\ldots ;x_{N}^{⊤}\right]\in R^{N\times d}$, $y={\left[y_{1},\ldots ,y_{N}\right]}^{⊤}$. After mean-centering, the closed-form solution is given by:

$$\hat{\beta }={\left(X^{T}X+\lambda I\right)}^{-1}X^{T}Y$$

This model directly estimates the linear response of compaction degree to attitude angles and their short-term statistics. The regularization parameter λ suppresses the amplification effect of collinearity among $\left(\theta ,\phi ,\psi \right)$ and their derivatives, thereby enhancing generalization stability.

#### 2.5.2. XGBoost Algorithm

The relationship between attitude features and compaction degree often exhibits nonlinearity and threshold effects. For example, when $\left|\dot{\phi }\right|$ exceeds a certain magnitude, its marginal effect on compaction degree may change abruptly. To capture such patterns, the XGBoost regression model was employed, the core of which is an additive ensemble of trees:

$${\hat{y}}_{i}=\sum_{k=1}^{K}f_{k}\left(x_{i}\right),f_{k}\in F$$

Here, $f_{k}$ denotes the k-th CART regression tree, K is the total number of regression trees, and F represents the space of all possible regression trees. By adopting the squared loss and applying a second-order Taylor expansion to the objective function at the t-th iteration, we obtain:

$$L^{\left(t\right)}\approx \sum_{i=1}^{N}\left[g_{i}\;f_{t}\left(x_{i}\right)+\frac{1}{2}h_{i}\;f_{t}{\left(x_{i}\right)}^{2}\right]+\Omega \left(f_{t}\right),$$

Here, $g_{i}=\partial_{\hat{y}}\frac{1}{2}{\left(y_{i}-{\hat{y}}_{i}^{\left(t-1\right)}\right)}^{2}=-\left(y_{i}-{\hat{y}}_{i}^{\left(t-1\right)}\right)$, $h_{i}=\partial_{\hat{y}}^{2}\frac{1}{2}{\left(y_{i}-{\hat{y}}_{i}^{\left(t-1\right)}\right)}^{2}=1$.

For each new leaf node j, the optimal weight is given by

$$w_{j}^{⋆}=-\;\frac{\sum_{i\in I_{j}}g_{i}}{\sum_{i\in I_{j}}h_{i}+\lambda_{t}}$$

where $I_{j}$ denotes the set of sample indices assigned to leaf j, and $\lambda_{t}$ is the regularization term for the leaf node. By learning splitting thresholds across features, XGBoost incrementally fits the attitude–compaction relationship. The regularization term Ω controls tree depth and the number of leaves, thereby preventing overfitting of noise patterns into the model.

#### 2.5.3. Random Forest Regression (RF)

To address construction disturbances and stochastic fluctuations in sensor data, a bagging ensemble strategy was adopted. Specifically, bootstrap sampling was applied to generate subsets $S_{b}$ from the training pairs $\left(x_{i},y_{i}\right)$. A regression tree $T_{b}$ was then trained on each subset, where at every node only a random sub-dimension of x was considered for selecting the splitting feature mmm and threshold τ, with the objective of maximizing the reduction in mean squared error:

$$\Delta \mathrm{SSE}=\sum_{i\in R}{\left(y_{i}-{\bar{y}}_{R}\right)}^{2}-\sum_{c\in \{L,R\}}\sum_{i\in R_{c}}{\left(y_{i}-{\bar{y}}_{R_{c}}\right)}^{2}$$

where R denotes the sample set at the current node, $R_{L}=\{i:\;x_{i,m}\leq \tau \},\;R_{R}=\{i:\;x_{i,m}>\tau \}$. The final prediction is obtained as the average of all trees:

$${\hat{y}}_{i}=\frac{1}{B}\sum_{b=1}T_{b}(x_{i})$$

where $T_{b}(x)$ denotes the prediction of the b-th tree. This process performs diversified splits on the raw attitude angles as well as derived features such as short-term activity, variance, and composite inclination, thereby reducing the high variance of individual trees through averaging and enhancing robustness of compaction prediction under small fluctuation scenarios.

### 2.6. Model Performance Evaluation

The model performance was comprehensively evaluated using the Root Mean Square Error (RMSE) and the coefficient of determination ($R^{2}$). Under these two indicators, a smaller RMSE and a larger R^2^ indicate a better fitting performance.

$$\begin{array}{c} \mathrm{RMSE}=\sqrt{\frac{1}{n}\sum_{i=1}^{n}\;{\left({\hat{c}}_{i}-c_{i}\right)}^{2}}\\ R^{2}={\left(\frac{cov(\hat{c},c)}{\sigma_{\hat{c}}\sigma_{c}}\right)}^{2} \end{array}$$

To enhance model robustness, a 5-fold cross-validation (CV) procedure was implemented during training. For each fold, model performance was evaluated in terms of RMSE and R^2^, and the mean ± standard deviation across folds (CV_RMSE, CV_R^2^) were reported. This strategy ensured stable and unbiased model generalization.

## 3. Results

### 3.1. Characteristics of Raw Attitude Signals During the Compaction Process

**Figure 4:**
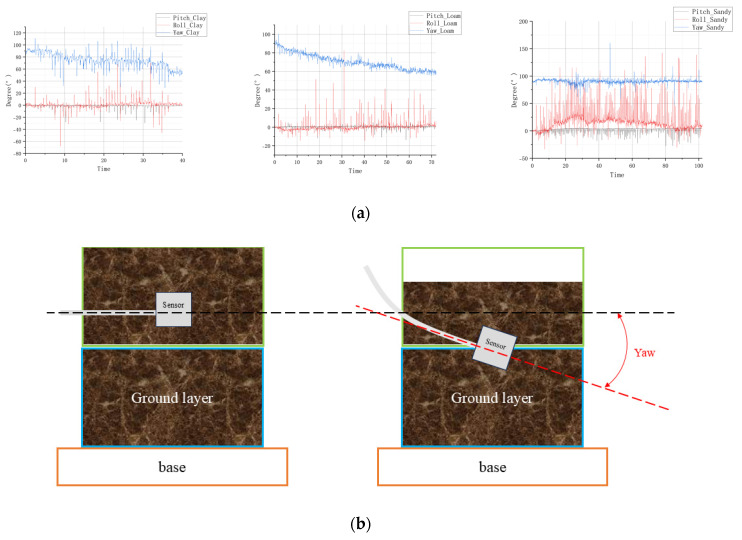
(**a**) Attitude variation curves during compaction for the three soil types: clay, loam and sand. (**b**) Schematic diagram indicating yaw angle’s change under manual compaction.

As shown in Figure 4, both transient and baseline-level variations can be observed throughout the compaction process. During the compaction process, vertical settlement of the soil induces small but measurable rotations of the embedded sensor due to heterogeneous stiffness distribution and lateral stress redistribution. Specifically, rotation about the vertical (yaw) axis represents the degree of lateral confinement as the soil densifies. This rotation response provides an indirect yet sensitive indicator of the internal deformation pattern. The effect is most pronounced in cohesive soils, where non-uniform compaction produces local torque moments; moderate in loam, which exhibits mixed plastic–frictional behavior; and weakest in sandy soils, whose deformation tends to be more homogeneous. This physical interpretation, supported by the observed attitude trajectories, explains the distinct response amplitudes among the three tested soil types. At the transient level, each hammer impact is accompanied by a directional non-zero mean disturbance, representing instantaneous changes in internal attitude induced by the impact force. At the baseline level, a gradual trend in attitude evolution can be identified as compaction progresses.

For clay and loam, pronounced variations in Euler angles are observed over time. Specifically, the baseline of pitch and yaw angles exhibits a decreasing trend during compaction, whereas the roll angle shows the opposite tendency. In contrast, the angular variations in sand compaction are relatively minor. Among these, the yaw angle corresponds to vertical settlement, the pitch angle reflects lateral tilting, and the roll angle represents transverse rotation. Yaw is particularly sensitive to the compaction trend, while pitch and roll variations are partly attributable to the instability of manual compaction, such as uneven loading and unstable lateral posture of the mold, which warrants further analysis.

### 3.2. Comparison and Analysis of Attitude Signal Filtering Results

**Figure 5:**
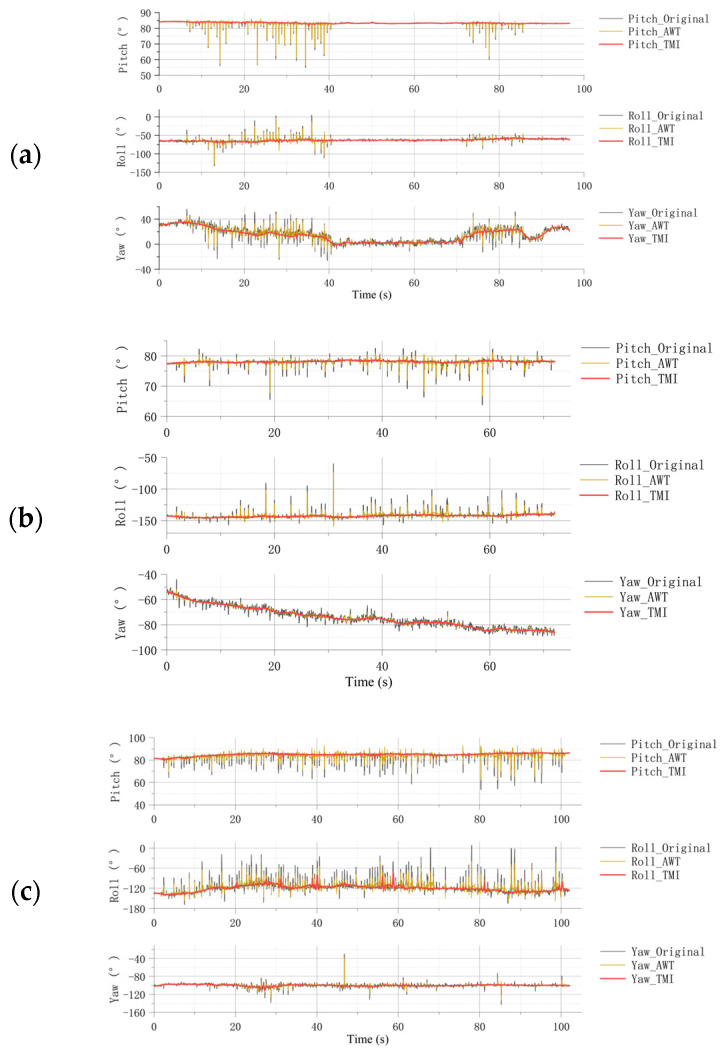
Comparison of raw attitude angle data and filtered attitude angle data using two methods during the compaction process. (**a**) Clay; (**b**) loam; (**c**) sand.

Figure 5 presents a comprehensive comparison between the raw signals and two filtering approaches. For all three soil types (clay, loam, and sand), the raw attitude angle signals during compaction exhibit pronounced transient disturbances and high-frequency fluctuations. In contrast, the curves processed by the two filtering methods are smoothed to varying degrees, with the TMI method demonstrating a clear advantage in preserving overall trends. As shown in Figure 5, although the TMI-filtered curves still exhibit certain numerical deviations relative to the raw signals, they closely follow the baseline variation trend of the original data. This prevents excessive attenuation of low-frequency components and ensures that the global evolution pattern of the compaction process is retained. Table 2 shows the Euler angle change during all the experiments.

Table 3 further highlights the differences between the two filtering algorithms. In most cases, the AWT method yields smaller RMSE values. For example, in the clay specimen, the RMSE of the pitch angle decreases from 3.362 with TMI to 1.069 with AWT, indicating superior amplitude recovery accuracy of AWT. However, in terms of smoothness and ΔSmoothness indices, the TMI method significantly reduces signal fluctuations. This effect is particularly evident in the roll and yaw angles, where TMI substantially suppresses high-frequency noise. For instance, in the sand specimen, the smoothness of the roll angle decreases to only 0.1054 with TMI, whereas AWT remains as high as 24.81, showing a clear disparity.

Overall, AWT tends to more closely approximate the raw signal in terms of numerical accuracy, but it may lose portions of baseline information while suppressing transient disturbances, thereby limiting its ability to represent the long-term compaction trend. By contrast, although TMI exhibits larger RMSE values, it produces smoother outputs while preserving baseline variation trends, making it better suited for analyzing the macroscopic evolution of the compaction process. For subsequent tasks such as soil classification and discrimination based on compaction curve morphology, the advantages of TMI are of greater practical value.

### 3.3. Feature Sensitivity Analysis

A sensitivity analysis of various combinations of attitude-based features was conducted using the Pearson correlation coefficient:

$$r_{X_{i},Y}=\frac{\sum_{k=1}^{n}\left(x_{ik}-{\bar{x}}_{i})(y_{k}-\bar{y}\right)}{\sqrt{\sum_{k=1}^{n}{\left(x_{ik}-{\bar{x}}_{i}\right)}^{2}}\sqrt{\sum_{k=1}^{n}{\left(y_{k}-\bar{y}\right)}^{2}}}$$

where ${\bar{x}}_{i}=\frac{1}{n}\Sigma_{k=1}^{n}x_{ik}$ is the mean of the target variable Y and $\bar{y}=\frac{1}{n}\Sigma_{k=1}^{n}y_{k}$. A value of $r_{X_{i},Y}$ closer to +1 indicates a strong positive correlation between the feature and compaction degree, whereas values closer to –1 indicate a strong negative correlation. Values near zero suggest a weak relationship between the feature and compaction degree.

**Figure 6:**
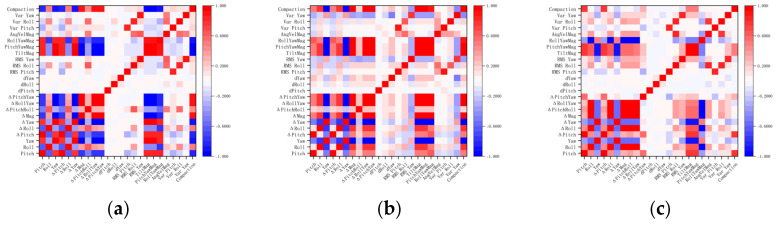
Heatmap analysis of different features with respect to soil compaction degree. (**a**) Clay; (**b**) loam; (**c**) sand.

Based on this analysis, the feature–compaction relationships are visualized in the heatmap shown in Figure 6. It can be observed that the yaw angle, as well as its combinations with pitch and roll angles, exhibits a strong correlation with the degree of compaction.

### 3.4. Construction and Validation of Compaction Regression Prediction Models

**Figure 7:**
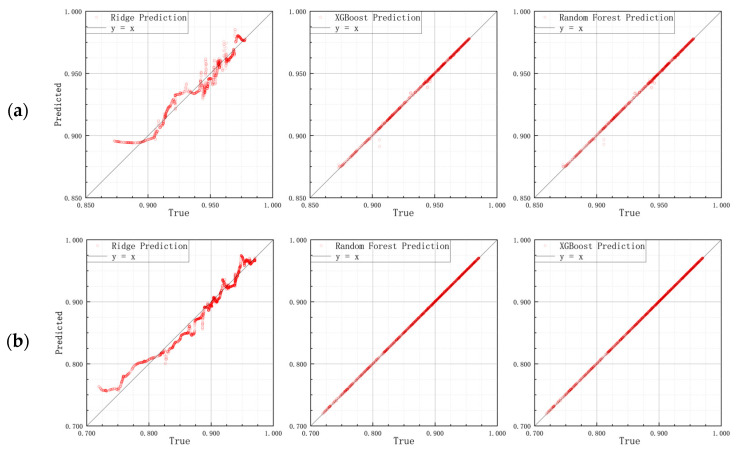

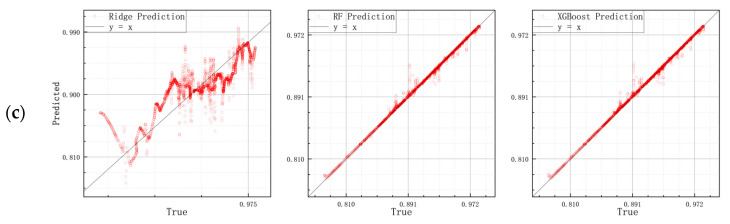
Prediction performance of soil compaction degree using Ridge Regression, RF, and XGBoost models for (**a**) clay, (**b**) loam, and (**c**) sand specimens.

Based on the experimental design, feature–compaction prediction models were developed, and their results were analyzed as follows:

As shown in Figure 7, the prediction performance of compaction degree by Ridge Regression, Random Forest, and XGBoost models across different soil specimens (clay, loam, and sand) was generally satisfactory, with the overall fitted curves exhibiting strong agreement with the y = x reference line. A comparative analysis reveals that Ridge Regression demonstrated lower stability, while Random Forest effectively captured nonlinear relationships with relatively concentrated fitting points, though slight dispersion remained in the medium-to-high compaction range. In contrast, XGBoost consistently achieved the highest predictive accuracy for all three soil types, with predictions in the high compaction region (>0.90) nearly coinciding with the measured values.

Table 4 further quantifies these findings. In terms of RMSE and $R^{2}$, Ridge Regression was constrained by its linear assumption, resulting in overall lower accuracy compared with the two nonlinear approaches. Random Forest substantially improved fitting performance in most ranges but still exhibited fluctuations for individual predictions. XGBoost achieved the lowest RMSE and highest $R^{2}$ across all soil types, with $R^{2}$ exceeding 0.995 in the high compaction range.

## 4. Discussion

The present study, based on an independently developed embedded attitude sensor, verified the effectiveness of posture as an indicator for monitoring the compaction degree during the compaction process of the 3 typical roadbed soil, and XGBoost, owing to its gradient boosting mechanism and feature interaction capability, is better suited to capture higher-order nonlinear couplings among features. From an engineering perspective, the high compaction range is typically critical for construction quality control. Therefore, the superior performance of XGBoost in this region highlights its greater practical applicability, offering more reliable real-time predictions and assessments of compaction degree for subgrade construction.

While the rigid 4 cm aluminum–nylon housing may locally alter the soil’s stress distribution during compaction, the proposed method interprets relative attitude variations rather than absolute stress responses, this geometric influence does not compromise the validity of the measurement. As a potential improvement, future work may investigate miniaturized or flexible packaging to further mitigate this effect. Additionally, The long-term service life and weather resistance of the embedded sensors were not experimentally assessed in this study. The aluminum–nylon composite casing, however, was selected for its resistance to mechanical stress, corrosion, and moisture. Future field trials will evaluate durability under cyclic loading, temperature, and humidity variations. The current prototype sensor costs approximately USD 50–100 per unit. With large-scale manufacturing and material optimization, the cost is expected to decrease substantially.

Although this study investigated three representative soil types (clay, loam, and sand), these categories were intentionally chosen to cover distinct mechanical behaviors. The proposed methodology, which relies on relative attitude variation rather than absolute soil stress, is general in principle and can be extended to mixed or natural field soils. Future work will validate the approach using in situ and heterogeneous soil samples to assess its broader applicability.

Although this study verified the correlation between embedded attitude sensor measurements and soil compaction degree under cyclic manual impact compaction, it remains limited to laboratory-scale Marshall specimens and does not account for other compaction methods. Furthermore, only three soil types with fixed quantitative proportions were tested, which demonstrated preliminary feasibility but fell short of meeting the data requirements of real-world engineering practice.

The current prototype sensor has not yet undergone long-term field deployment; thus, potential long-term drift, fatigue, and environmental degradation effects remain to be evaluated. These aspects will be systematically addressed in future studies involving field compaction and extended environmental exposure to ensure stable real-world performance. Future work will focus on experiments under actual field conditions, incorporating a broader variety of subgrade soils and more diverse compaction methods. Large-scale validation and analysis will be necessary to better align with engineering practice and to further enhance the generalization capability of compaction prediction models based on embedded attitude sensors. In practical applications, multiple sensors can be strategically installed at key subgrade depths or lateral positions to complement conventional compaction indicators such as the Compaction Meter Value (CMV) or Compaction Control Coefficient (CCC). Integrating these datasets through data-fusion algorithms could provide a spatially distributed and temporally continuous assessment of roadbed compaction quality, thereby forming the foundation for an intelligent compaction monitoring network. Collectively, the findings presented herein provide laboratory-scale proof of concept for this attitude-based sensing approach and a clear technical pathway toward robust, field-ready implementation.

## 5. Conclusions

This study conducted experiments and analyses on a dynamic identification method for subgrade soil compaction based on embedded attitude sensors. The main conclusions are as follows:

- By designing an embedded attitude sensor and implementing attitude estimation based on acceleration and magnetic field data, it became possible to acquire real-time signals of internal attitude changes in subgrade soils during compaction, thereby providing a novel monitoring approach for dynamic identification of compaction degree. Experimental analysis indicated that, under the installation configuration adopted in this study, the yaw angle was the most sensitive to vertical settlement, with variations of −36.3°, −31.4°, and −4.2° in clay, loam, and sand samples, respectively—substantially higher than those of pitch and roll angles, whose maximum values were only 5.85° and 8.10°. This demonstrates that the yaw angle can serve as a core indicator for compaction identification, while pitch and roll provide complementary information on lateral and rotational tendencies.
- To address transient disturbances caused by impacts, two filtering strategies—AWT and TMI—were compared. Although the TMI method yielded relatively larger RMSE values, it exhibited superior smoothness and effectively preserved baseline trends. For example, in the roll angle of sand samples, the smoothness index was reduced to 0.1054 with TMI, whereas AWT remained as high as 24.81. These results indicate that TMI is more suitable for capturing long-term compaction patterns, particularly advantageous in macro-trend analysis and soil type discrimination.
- A feature dataset was extracted from the attitude data, and correlation analyses were performed between these features and compaction degree. Ridge regression, random forest, and XGBoost models were employed to establish mapping relationships. The results showed that ridge regression was limited by its linear assumption, resulting in lower predictive accuracy (R^2^ = 0.7092 for sand). In contrast, both random forest and XGBoost significantly improved the fitting performance. XGBoost achieved R^2^ values exceeding 0.999 for clay and loam and maintained 0.997 for sand, with RMSE values on the order of 10^−4^–10^−3^. At high compaction levels (>0.90), the predictions of XGBoost nearly overlapped with the measured values, highlighting its potential for practical application in critical stages of construction quality control.
- The present work serves as a laboratory-scale proof of concept. The limitation lies in the fact that the current road subgrade and pavement environments vary, requiring potential different technical index, i.e., differentiated selection of sensors. Additionally, since the sensor casings are made of relatively rigid materials, they are bound to have an impact on the stress distribution within the soil layer. Therefore, after the sensors are buried in the roadbed soil, the reliability and tolerance of the soil layer need to be further verified.

## Figures and Tables

**Table 1:** List of physical parameters of the three soil types.

Subgrade Soil Type	Silty Clay	Silty Loam	Silty Sand
Maximum Dry Density (g/cm^3^)	1.93	2.17	2.03
Optimum Water Content (%)	13.7	12.6	9.4

**Table 2:** List of Euler Angle maximum changes of the three soil types during experiments.

Subgrade Soil Type	Silty Clay	Silty Loam	Silty Sand
Pitch	0.06	0.62	5.85
Roll	4.05	3.37	8.10
Yaw	−36.3	−31.43	−4.2043

**Table 3:** Comparison of the two transient disturbance filtering methods.

Soil Type	Signal	Method	RMSE	Smoothness	ΔSmoothness
Clay	Pitch_deg	TMI	3.362	0.001297	−1.982
		AWT	1.069	1.719	−0.2645
	Roll_deg	TMI	9.007	0.005152	−11.41
		AWT	4.126	6.37	−5.043
	Yaw_deg	TMI	8.01	0.01309	−10.53
		AWT	5.322	4.447	−6.096
Loam	Pitch_deg	TMI	1.174	6.203 × 10^−5^	−0.3805
		AWT	0.6269	0.2749	−0.1056
	Roll_deg	TMI	5.495	2.284 × 10^−4^	−5.481
		AWT	2.946	3.744	−1.737
	Yaw_deg	TMI	1.936	8.085 × 10^−5^	−0.7847
		AWT	1.701	0.1256	−0.6591
Sandy	Pitch_deg	TMI	3.856	0.001392	−1.978
		AWT	2.5	1.444	−0.5354
	Roll_deg	TMI	17.22	0.1054	−46.19
		AWT	11.46	24.81	−21.48
	Yaw_deg	TMI	3.771	−0.001298	−2.539
		AWT	2.365	1.272	−1.269

**Table 4:** Comparison of RMSE and $R^{2}$ values for the prediction performance of the three models.

Soil Type	Model	RMSE	R^2^	CV RMSE	CV R^2^
Clay	Ridge	0.0063	0.9441	0.0008	0.9989
	XGBoost	0.0006	0.9994	0.0008	0.999
	RF	0.0006	0.9994	0.0062	0.9442
Loam	Ridge	0.0104	0.9715	0.0003	1
	XGBoost	0.0002	1.0000	0.0003	1
	RF	0.0002	1.0000	0.0104	0.9713
Sandy	Ridge	0.0245	0.7092	0.0026	0.9967
	XGBoost	0.0025	0.9970	0.0025	0.9968
	RF	0.0025	0.9971	0.0238	0.7123

## Data Availability

The raw data supporting the conclusions of this article will be made available by the authors on request.

## Abbreviations

The following abbreviations are used in this manuscript:

TMI	Transient Masking Interpolation
AWT	Asymmetric Wavelet Thresholding
RMSE	Root Mean Square Error
SWT	Stationary Wavelet Transform
MAD	Median Absolute Deviation
RR	Ridge Regression
RF	Random Forest
